# Supplementary material for: Identification and Functional Analysis of a Lysozyme Gene from Coridius chinensis (Hemiptera: Dinidoridae)
Source: Biology (Basel). 2021 Apr 14;10(4):330. doi: 10.3390/biology10040330 (PMC8071013; doi:10.3390/biology10040330)
Supplement: Supplementary file 1 [file biology-10-00330-s001.zip › biology-1167104-supplementary.pdf]

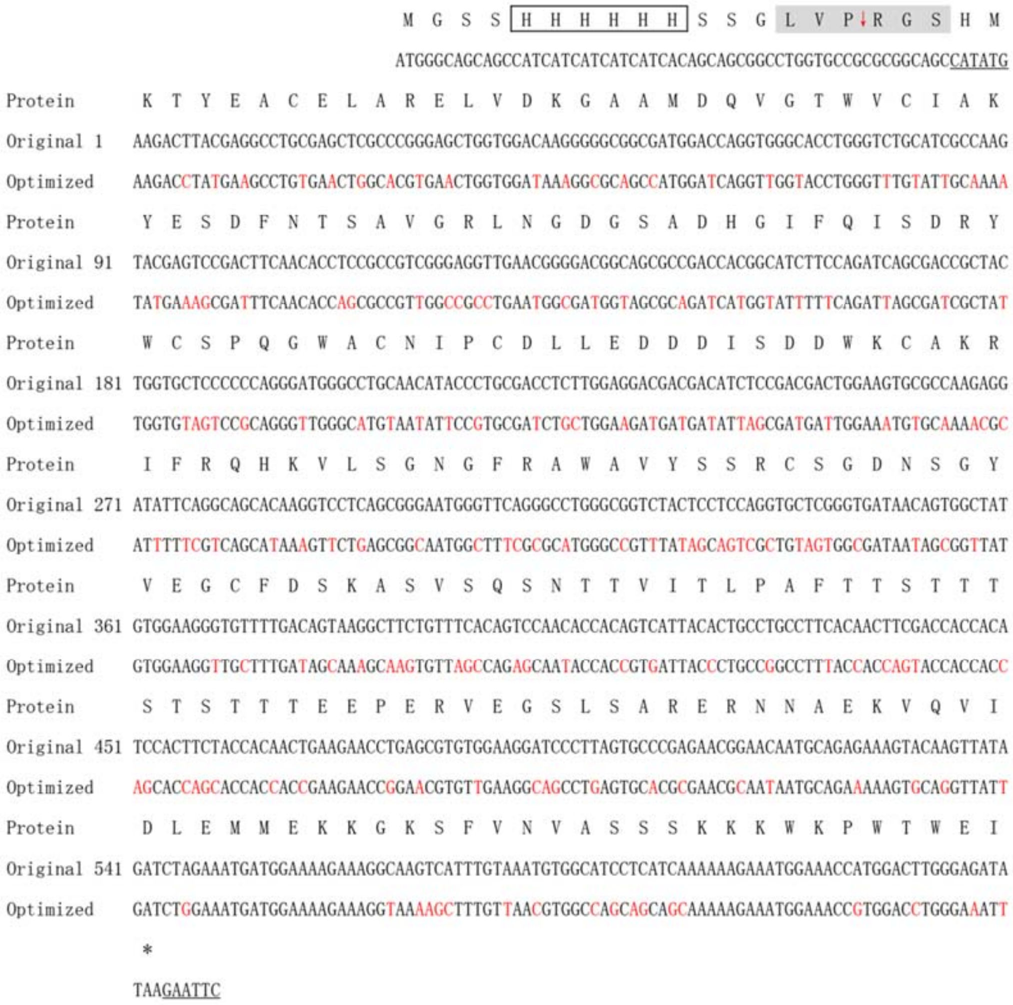

Figure S1. Optimized nucleotide and amino acid sequences of the mature CcLys2. The total length of the optimized gene is 633 bp, and red marks the mutated bases. The recognition sites for *Nde* I and *Eco*R I are underlined, and the red arrow marks the thrombin cleavage site. The N-terminal of the amino acid sequence contains a 6×His-tag. The asterisk marks the stop codon.

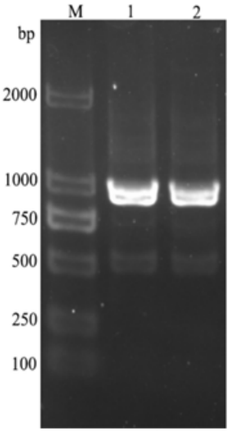

Figure S2. Identification of the recombinant expression vector by PCR. M: DL2000 DNA marker; Lanes 1 and 2: the *CcLys2* gene with a part of the pET-28b(+) vector sequence.

Table S1. Species name, protein name and GenBank accession number for multiple sequence alignment and phylogenetic analysis.

Protein name	Species name	GenBank accession number
CcLys2	*Coridius chinensis*	QPB70596
CcLLP3	*Coridius chinensis*	MW250790
HEWL	*Gallus gallus domesticus*	1LSZ_A
HLYZ1	*Homo sapiens*	NP_000230
PsLys	*Plautia stali*	BBE08150
HhLys	*Halyomorpha halys*	KAE8573139
TcLys1	*Tribolium castaneum*	ACV32411
TcLys2	*Tribolium castaneum*	ACV32412
HaLLP1	*Harmonia axyridis*	AIZ72680
HaLLP2	*Harmonia axyridis*	AIZ72679
HaLys3	*Harmonia axyridis*	AIZ72681
HaLys4	*Harmonia axyridis*	AIZ72682
AgLYSC1	*Anopheles gambiae*	Q17005
AgLys c-6	*Anopheles gambiae*	AAY21238
AgLys c-8	*Anopheles gambiae*	AAY21241
CqLys	*Culex quinquefasciatus*	XP_001843429
FoLys	*Frankliniella occidentalis*	KAE8737425
NlLys	*Nilaparvata lugens*	AGK40894
BmLZ	*Bombyx mori*	NP_001037448
BLLP1	*Bombyx mori*	ABP52099
ALLP1	*Antheraea mylitta*	ABP52098
CfLys3	*Coptotermes formosanus*	AFZ78838
SeLys3	*Spodoptera exigua*	AKJ54522
OfLys5	*Ostrinia furnacalis*	AGV28584

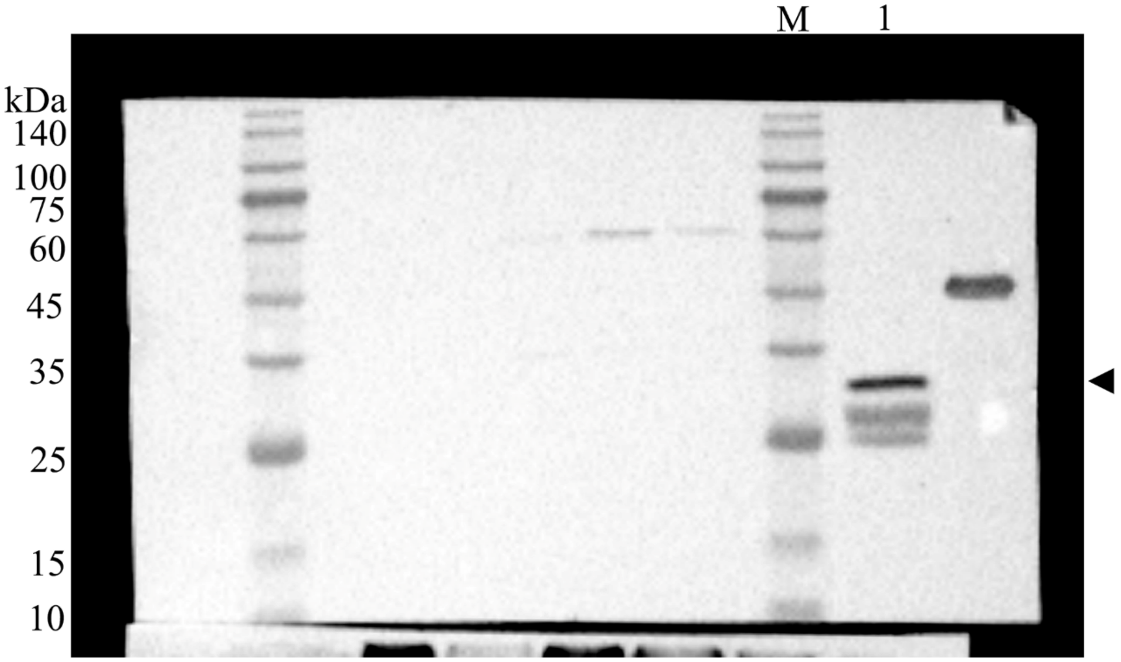

Figure S3. M: Protein molecular weight marker (10-140 kDa). Lane 1: blotting band of fusion CcLys2. The arrow indicates the band of fusion CcLys2.
